# Supplementary material for: Evaluating Rhizobacterial Antagonists for Controlling Cercospora beticola and Promoting Growth in Beta vulgaris
Source: Microorganisms. 2024 Mar 27;12(4):668. doi: 10.3390/microorganisms12040668 (PMC11052011; doi:10.3390/microorganisms12040668)
Supplement: Supplementary file 1 [file microorganisms-12-00668-s001.zip › microorganisms-2896619-supplementary.pdf]

Supplementary Information (8 pages):

- **Five supplementary tables**
- **Four supplementary figures**
- **References of Tables S1 and S2**

Table S1. Oligonucleotide sequences (in the 5' to 3' direction) were employed for the amplification of genes encoding lipopeptides from the DNA of bacterial isolates.

Lipopeptides	Primers	Primer sequences	PCP length (bp)	Annealing T°	References
Bacillomycin	Bacc1F<br>Bacc1R	GAAGGACACGGCAGAGAGTC<br>CGCTGATGACTGTTCATGCT	875	60 °C	[1]
Fengycin	Fend1F<br>Fend1R	TTTGGCAGCAGGAGAAGTT<br>GCTGTCCGTTCTGCTTTTTC	964	62 °C	[1]
Iturin	Itup1F<br>Ituo2R	AGCTTAGGGAACAATTGTCATCGGGGCTTC<br>TCAGATAGGCCGCCATATCGGAATGATTCG	2000	45 °C	[2]
Surfactin	P17<br>P18	ATGAAGATTTACGGAATTTA<br>TTATAAAAGCTCTTCGTACG	675	53 °C	[3]

Table S2. Diverse biochemical analyses were performed, encompassing both the aspect of revelation and the evaluation of activity indices.

Biochemical test	Media reference	Revelation aspect	Activity index evaluation	Activity index evaluation reference
**Cellulase**	[4]	clear circular area around the bacteria colony	(Clear zone+ colony diameter (mm)/colony diameter (mm))	[5]
**Pectinase**	[6]	clear circular area around the bacteria colony	(Clear zone+ colony diameter (mm)/colony diameter (mm))	[5]
**Amylase**	[7]	clear circular area around the bacteria colony	(Clear zone+ colony diameter (mm)/colony diameter (mm))	[8]
**Protease**	[4]	clear circular area around the bacteria colony	(Clear zone+ colony diameter (mm)/colony diameter (mm))	[9]
**Chitinase**	[10]	clear circular area around the bacteria colony	(Clear zone+ colony diameter (mm)/colony diameter (mm))	[5]
**Phosphate solubilisation**	[5]	clear circular area around the bacteria colony	(Clear zone+ colony diameter (mm)/colony diameter (mm))	[11]
**HCN**	[12]	change of coloration from yellow to reddish-brown	(-) negative; light brown (+); brown (++) dark brown (+++)	[13]
**AIA**	[14]	change of coloration from yellow to red	(-) negative; light red (+); red (++) dark red (+++)	-

Table S3. Application of treatments with varying concentrations against C. beticola in the field experiment.

Treatments	Active ingredient	Concentration g/(l-kg)	Active ingredient/ha	Code
SCORE 250 EC (SYNGENTA)	Difenoconazole	250	125	DF
BGH 1-6	*Pantoea sp.*	1x10^8 CFU/ml	4x10^12 CFU	BGH 1-6
BGH 2-2	*Serratia sp.*	1x10^8 CFU/ml	4x10^12 CFU	BGH 2-2
BGH 1-3	*Serratia sp.*	1x10^8 CFU/ml	4x10^12 CFU	BGH 1-3
BGH 2-7	*Bacillus sp.*	1x10^8 CFU/ml	4x10^12 CFU	BGH 2-7
untreated control				UC

Table S4. The impact of bacterial inoculation on the growth of sugar beet plants in a greenhouse experiment.

Bacterial isolates	Root dry weight[a] (g)	Shoot dry weight[a] (g)	Root length[a] (mm)	shoot length[a] (mm)	Gain of root lenght (%)	Gain of shoot length[b] (%)	Gain of Shoot dry weight[b] (%)	Gain of root dry weight[b] (%)	Total gain of root dry weight[b] (%)	Root hair development[c]
**BGH 1-5**	1.09±0.04	1.70±0.02	44.13±1.79	22.28±0.30	72.70%	389%	78%	107%	86%	+
**G1b**	1.22±0.02	1.49±0.03	31.13±1.26	18.74±0.65	45.30%	245%	56%	131%	81%	++
**BGH 2-1**	1.06±0.072	1.62±0.10	38.12±4.93	21.81±0.80	69.10%	322%	70%	101%	79%	+
**BGH 4-1**	0.93±0.07	1.72±0.02	17.68±0.42	23.01±0.40	78.40%	96%	80.50%	77%	77%	++
**BGH 1-6**	1.15±0.17	1.44±0.13	25.12±9.46	18.58±2.06	44.00%	178%	51%	118%	73%	+
**BGH 2-3**	1.11±0.02	1.36±0.05	47.43±1.23	16.83±0.45	30.50%	425%	43%	111%	65%	++
**G3f**	1.12±0.05	1.34±0.10	34.59±0.83	16.13±0.66	25.10%	283%	41%	113%	64%	+++
**G2c**	1.12±0.06	1.22±0.30	26.48±0.95	15.29±2.55	18.50%	193%	29%	114%	57%	+++
**G2b**	0.94±0.07	1.31±0.02	16.29±1.12	16.25±0.39	26.00%	80%	37%	79%	50%	++++
**BGH 2-2**	0.89±0.07	1.26±0.10	18.01±0.85	15.9±0.74	23.30%	99%	33%	70%	44%	++
**BGH 1-3**	0.82±0.06	1.26±0.05	14.23±0.23	16.0±0.43	24.00%	58%	32%	56%	39%	+
**BGH 2-7**	0.56±0.03	1.51±0.15	9.43±1.03	19.675±2	52.50%	4%	59%	6%	38%	+
**G1d**	0.76±0.07	1.22±0.06	13.5±0.4	15.73±0.76	22.00%	49%	28%	44%	32%	++++
**G3d**	0.77±0.12	1.20±0.060	13.33±1.03	15.38±0.77	19.30%	48%	26%	47%	32%	++
**BGH 2-5**	0.74±0.07	1.19±0.04	14.36±0.50	15.28±0.86	18.50%	59%	25%	41%	29%	++
**G1a**	0.50±0.04	1.28±0.07	8.52±0.47	16.60±0.27	28.70%	-6%	34%	-4%	19%	++
**G3c**	0.50±0.01	1.04±0.05	8.80±0.47	13.77±1.16	6.80%	-3%	10%	-4%	3%	++++
**TNT**	0.52±0.01	0.95±0.13	9.03±0.70	12.91±0.56	0%	0%	0%	0%	0%	+++
**G4a**	0.61±0.02	0.87±0.18	11.45±0.47	12.60±0.26	-2.30%	27%	-8%	16%	-1%	++++

[a] The values represent the mean of three independent assay replicates, expressed as the mean ± standard error, with units in grams (g) and millimeters (mm).

[b] Percentages are derived by comparing inoculated versus non-inoculated samples.

[c] The gradation of responses for the trait of root hair development, ranging from strong to weak, is denoted as (+ + ++), (+ + +), (+ +), and (+).

Figure S1.

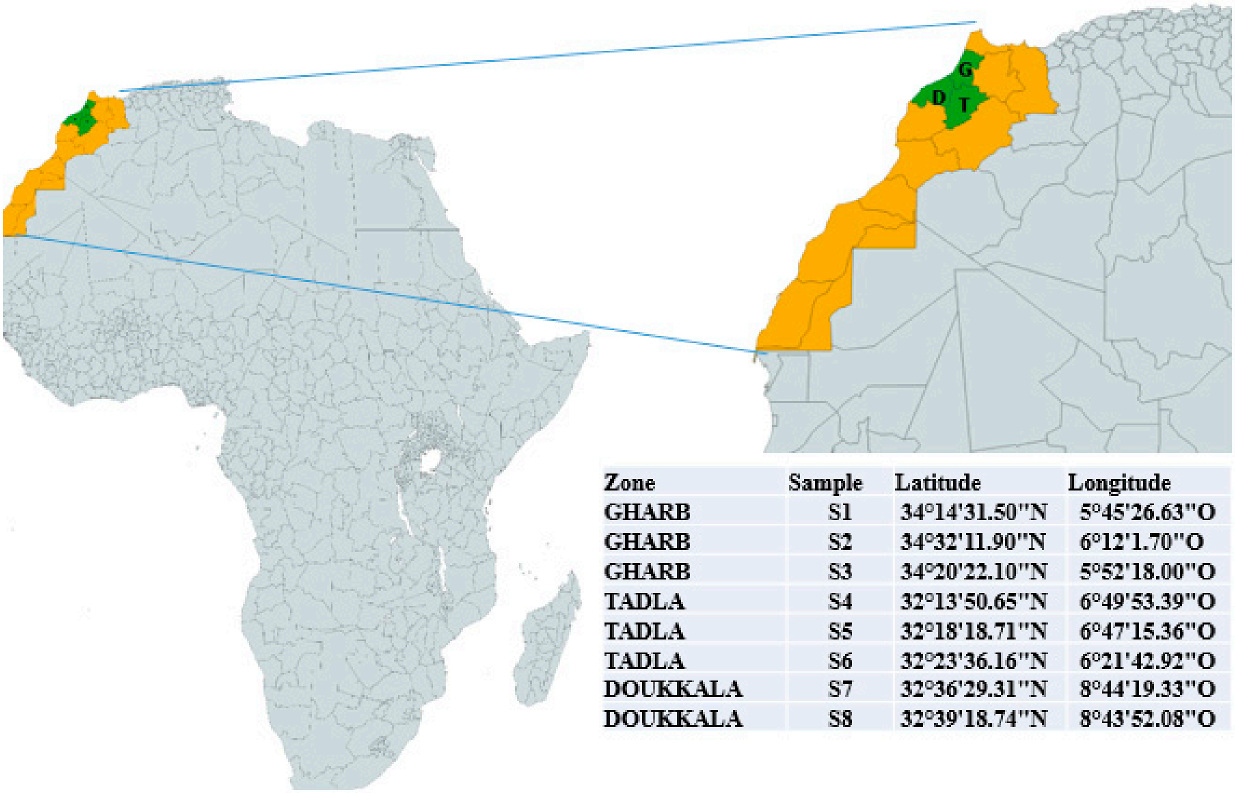

Zone	Sample	Latitude	Longitude
GHARB	S1	34°14'31.50"N	5°45'26.63"O
GHARB	S2	34°32'11.90"N	6°12'1.70"O
GHARB	S3	34°20'22.10"N	5°52'18.00"O
TADLA	S4	32°13'50.65"N	6°49'53.39"O
TADLA	S5	32°18'18.71"N	6°47'15.36"O
TADLA	S6	32°23'36.16"N	6°21'42.92"O
DOUKKALA	S7	32°36'29.31"N	8°44'19.33"O
DOUKKALA	S8	32°39'18.74"N	8°43'52.08"O

Figure S1. A map showing the 6 sites that have been sampled in Morocco in three regions: G, Gharb; D, Doukkala; and T, Tadla.

Figure S2.

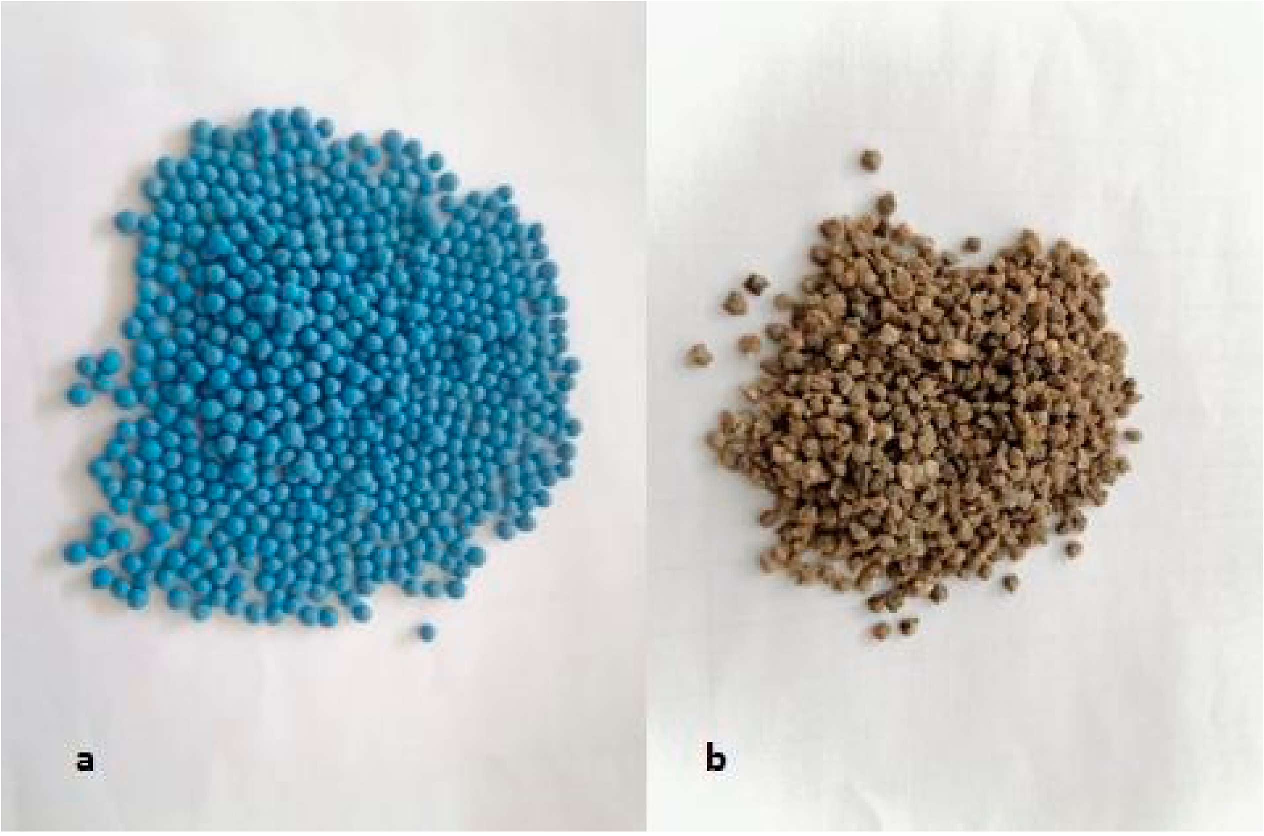

Figure S2. Panel a displays seeds of sugar beet with a coating, whereas Panel b illustrates seeds that have been washed to eliminate the coated reagents.

Figure S3.

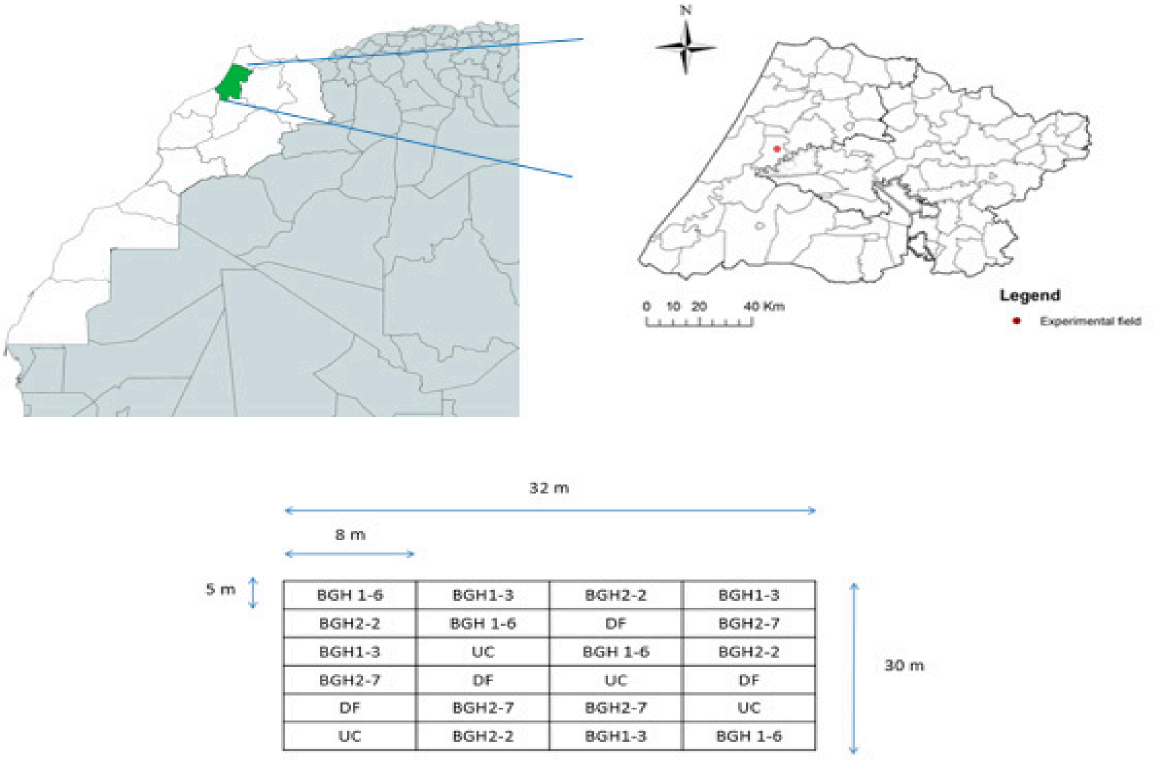

Figure S3. The field trial site's location and the experimental setup, including treatments, are shown. The four bacterial isolates (BGH1-6, *Pantoea* sp.; BGH 2-2, *Serratia* sp.; BGH 2-7, *Bacillus* sp.; and BGH 1-3, *Serratia* sp.), along with DF (Difenoconazole) and UC (untreated control), were employed. The experiment included four replicates. The dimensions of the plots are presented in meters.

Figure S4.

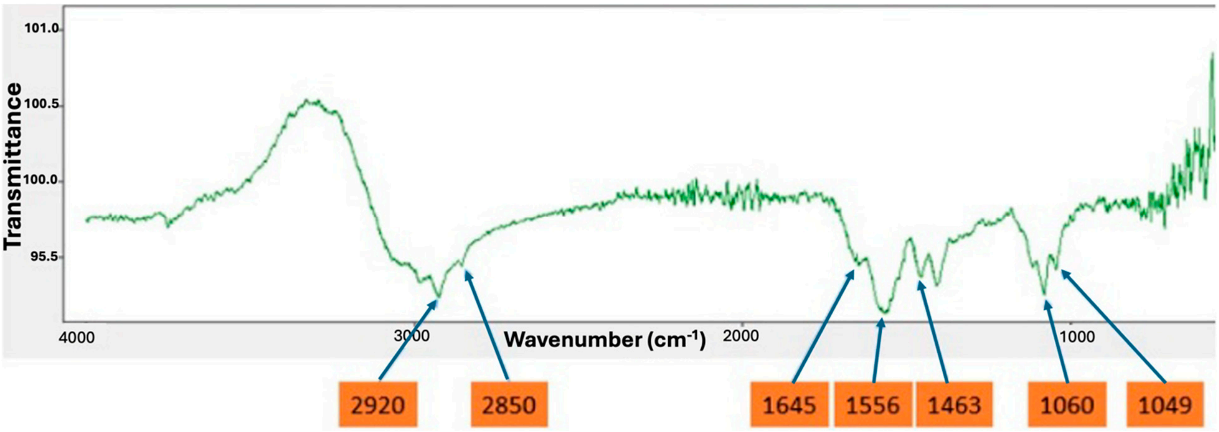

Figure S4. Fourier Transform Infrared (FTIR) Spectroscopy used to perform qualitative and quantitative analysis the bacterial isolate BGH2-2 supernatant.

Figure S5.

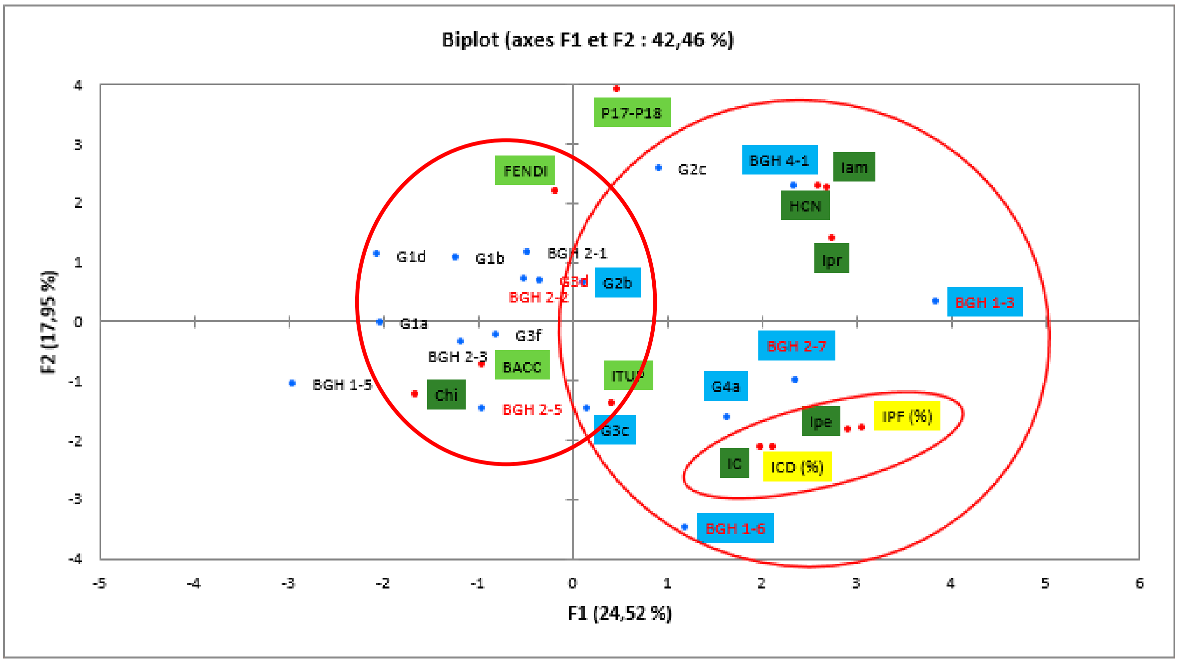

Figure S4**.** Principal components analysis was conducted for the simultaneous assessment of hydrolytic enzyme production, bacterial antagonism, and the presence of lipopeptide encoding genes (ipe: pectinase index; IC: cellulase index; Iam: amylase index; IPR: protease index; hcn: hydrogen cyanide production). Bacterial isolates highlighted in red exhibited a high inhibition rate in dual culture, while those with a blue background demonstrated a high indirect inhibition rate.

References used in Tables S1 abd S2

1. Ramarathnam, R.; Bo, S.; Chen, Y.; Fernando, W.G.D.; Xuewen, G.; De Kievit, T. Molecular and biochemical detection of fengycin-and bacillomycin D-producing Bacillus spp., antagonistic to fungal pathogens of canola and wheat. *Canadian Journal of Microbiology* **2007**, *53*, 901-911.
2. Dimkić, I.; Živković, S.; Berić, T.; Ivanović, Ž.; Gavrilović, V.; Stanković, S.; Fira, D. Characterization and evaluation of two Bacillus strains, SS-12.6 and SS-13.1, as potential agents for the control of phytopathogenic bacteria and fungi. *Biological Control* **2013**, *65*, 312-321.
3. Hsieh, F.C.; Li, M.C.; Lin, T.C.; Kao, S.S. Rapid detection and characterization of surfactin-producing Bacillus subtilis and closely related species based on PCR. *Current Microbiology* **2004**, *49*, 186-191, doi:10.1007/s00284-004-4314-7.
4. Syed-Ab-Rahman, S.F.; Carvalhais, L.C.; Chua, E.; Xiao, Y.; Wass, T.J.; Schenk, P.M. Identification of soil bacterial isolates suppressing different phytophthora spp. And promoting plant growth. *Frontiers in Plant Science* **2018**, *871*, doi:10.3389/fpls.2018.01502.
5. Ezrari, S.; Mhidra, O.; Radouane, N.; Tahiri, A.; Polizzi, G.; Lazraq, A.; Lahlali, R. Potential role of rhizobacteria isolated from citrus rhizosphere for biological control of citrus dry root rot. *Plants* **2021**, *10*, 1-26, doi:10.3390/plants10050872.
6. Etesami, H.; Alikhani, H.A.; Mirseyed Hosseini, H. Evaluation of halotolerant endophytic bacteria isolated from the halophyte suaeda for biological control of fungal rice pathogens. **2019**.
7. Dinesh, R.; Anandaraj, M.; Kumar, A.; Bini, Y.K.; Subila, K.P.; Aravind, R. Isolation, characterization, and evaluation of multi-trait plant growth promoting rhizobacteria for their growth promoting and disease suppressing effects on ginger. *Microbiological research* **2015**, *173*, 34-43.
8. Jamilah, I.T.; Meryandini, A.; Rusmana, I.; Suwanto, A.; Mubarik, N.R. Activity of proteolytic and amylolytic enzymes from Bacillus spp. isolated from shrimp ponds. *Microbiology Indonesia* **2009**, *3*, 4-4.
9. Pertiwiningrum, A.; Anggraini, F.D.; Fitrianto, N.A. Isolation and identification of bacterial protease enzyme of leather waste. *Journal of the Indonesian Tropical Animal Agriculture* **2017**, *42*.
10. Hsu, S.C.; Lockwood, J.L. Powdered Chitin Agar as a Selective Medium for Enumeration of Actinomycetes in Water and Soil1. *Applied Microbiology* **1975**, *29*, 422-426, doi:10.1128/aem.29.3.422-426.1975.
11. Rodrigues, A.A.; Forzani, M.V.; Soares, R.d.S.; Sibov, S.T.; Vieira, J.D.G. Isolation and selection of plant growth-promoting bacteria associated with sugarcane. *Pesquisa Agropecuária Tropical* **2016**, *46*, 149-158.
12. Lahlali, R.; Aksissou, W.; Lyousfi, N.; Ezrari, S.; Blenzar, A.; Tahiri, A.; Ennahli, S.; Hrustić, J.; MacLean, D.; Amiri, S. Biocontrol activity and putative mechanism of Bacillus amyloliquefaciens (SF14 and SP10), Alcaligenes faecalis ACBC1, and Pantoea agglomerans ACBP1 against brown rot disease of fruit. *Microbial Pathogenesis* **2020**, *139*, 103914-103914, doi:10.1016/j.micpath.2019.103914.
13. Modi, K.; Patel, P.; Parmar, K. Isolation, screening and characterization of PGPR from rhizosphere of rice. *International Journal of Pure & Applied Bioscience* **2017**, *5*, 264-270.
14. Yuttavanichakul, W.; Lawongsa, P.; Wongkaew, S.; Teaumroong, N.; Boonkerd, N.; Nomura, N.; Tittabutr, P. Improvement of peanut rhizobial inoculant by incorporation of plant growth promoting rhizobacteria (PGPR) as biocontrol against the seed borne fungus, Aspergillus niger. *Biological Control* **2012**, *63*, 87-97.
